# Sutureless Loop Enterostomy in Very Low and Extremely Low Birth Weight Infants

**DOI:** 10.7759/cureus.74296

**Published:** 2024-11-23

**Authors:** Yuki Muta, Akio Odaka, Seiichiro Inoue, Yuta Takeuchi

**Affiliations:** 1 Department of Hepato-Biliary-Pancreatic Surgery and Pediatric Surgery, Saitama Medical Center, Saitama Medical University, Kawagoe, JPN

**Keywords:** extremely low birth weight infants, focal intestinal perforation, meconium-related ileus, necrotizing enterocolitis, very low birth weight infants

## Abstract

Introduction

Sutureless enterostomy is used as an effective technique for constructing an enterostomy in very low and extremely low birth weight infants in Japan. Sutureless enterostomy is a separate type of enterostomy procedure for low birth weight infants. We adapted this technique and developed sutureless loop enterostomy (SLE), an approach without a skin bridge in which the intestinal wall is not sutured to the abdominal wall. This study aimed to compare SLE outcomes with those of sutureless enterostomy (SE) and the conventional procedure (C).

Methods

We retrospectively reviewed clinical records of 32 very low or extremely low birth weight infants who underwent enterostomy and classified the infants into three groups according to the procedure: SLE, SE, and C. We compared surgery-related items and enterostomy-related complications between the three groups.

Results

We found no significant differences in sex, age at surgery, or surgical blood loss. The operation time was significantly shorter in the SLE group than in the C group (P < 0.05). The number of postoperative complications was not significantly different in the SLE group compared with the other two groups.

Conclusion

Sutureless loop enterostomy is an effective, safe procedure for enterostomy in very low and extremely low birth weight infants.

## Introduction

With advancements in survival rates among very low and extremely low birth weight infants, there has been a rise in the number of enterostomy procedures performed to address conditions such as neonatal necrotizing enterocolitis (NEC), focal intestinal perforation (FIP), and meconium-related ileus (MRI) [1, 2]. In enterostomy, the intestine is usually anchored to the abdominal wall by suturing the seromuscular layer of the intestine to the peritoneum and/or abdominal wall fascia [3]. However, in low birth weight infants, this procedure is difficult because the intestinal wall is very thin and fragile [4], and enterostomy-related complications (e.g., necrosis, perforation, stenosis, fall, prolapse, surgical site infection, peristomal dermatitis, and parastomal hernia) frequently occur [5]. Furthermore, when these complications arise, many infants will require a subsequent surgical intervention to correct such adverse events, which can greatly affect the survival prognosis of these infants [6].

Since Ohashi et al. reported the effectiveness of sutureless enterostomy (SE) for extremely low birth weight infants in 2012, this technique has become widely used in Japan [7]. The SE technique developed by Ohashi et al. is a separate enterostomy that creates a skin bridge [6]. Other authors have reported on surgical techniques that fix the intestinal wall to the abdominal wall with gauze [4] or cyanoacrylate adhesive [8].

In cases that do not require intestinal resection, at our hospital we perform sutureless loop enterostomy (SLE), a procedure that does not create a skin bridge and does not suture the intestinal wall to the abdominal wall. The effectiveness of SLE is unknown because no studies have compared it with other enterostomy approaches in low birth weight infants. Therefore, the present study aimed to compare the efficacy and outcomes of SLE with those of SE and conventional enterostomy (C).

## Materials and methods

Study population

Thirty-six very low birth weight infants (i.e., birth weight <1500 g) and extremely low birth weight infants (i.e., birth weight <1000 g) underwent enterostomy at the Department of Hepato-Biliary-Pancreatic and Pediatric Surgery, Saitama Medical Center, Saitama Medical University, Saitama, Japan, between January 2013 and December 2020. For the purpose of this study, we excluded four patients who died because of an underlying disease before the enterostomy was closed. We retrospectively reviewed and analyzed the clinical records of the remaining 32 patients. Among the patients included in the study, eight had NEC, nine had FIP, nine had MRI, and six had other disease entities. We recorded gestational age, birth body weight, surgical procedure, surgery-related items, and enterostomy-related complications. Enterostomy-related complications that occurred less than 30 days after surgery were defined as early complications, and those that occurred 30 days or more after surgery, as late complications.

Patients underwent SLE as described below, SE as reported by Ohashi et al. or its modified procedure (the double-barreled-type enterostomy, which uses a similar procedure), or the conventional procedure in which the intestine is anchored to the abdominal wall. We classified the patients into three groups according to the type of enterostomy performed, i.e., SLE, SE, and C, and compared the groups. In patients who underwent multiple surgeries because of enterostomy complications, we included only the data from the initial procedure (the first surgery) in our analysis.

The study was approved by the Research Ethics Committee of Saitama Medical Center, Saitama Medical University (SMC2021-140). Consent was obtained from all patients at our institute in the form of an opt-out option on our institute website, and the need for informed consent was waived in view of the retrospective and observational nature of the study.

Surgical procedure of sutureless loop enterostomy (SLE)

The surgical procedure of SLE starts with a transverse incision in the upper abdomen, and, in most cases, dissection of the abdominal rectus muscle. If imaging has indicated an intestinal perforation or enlargement of the intestine, the laparotomy is performed above the lesion.

Next, a detailed intraperitoneal examination is performed. If the diagnosis is FIP, the intestinal perforation is used as the orifice of the enterostomy, and the tissue around the enterostomy orifice is evaluated by histopathology. If the diagnosis is MRI, the caliber of the intestine often is affected, so the enterostomy is constructed in the proximal intestine; histopathological examination of the tissue around the enterostomy orifice is performed in the same way as for FIP. If the diagnosis is NEC, SLE may be a suitable procedure for cases with a limited extent of damage, as it allows the necrotic or perforated segment to be exteriorized as the stoma. However, in cases of extensive NEC requiring resection of a large portion of the bowel, this technique may not be appropriate. If an intestinal perforation is present, the intraperitoneal cavity is irrigated with a large quantity of saline solution, and a Penrose or Blake drain is placed intraperitoneally.

**Figure 1:**
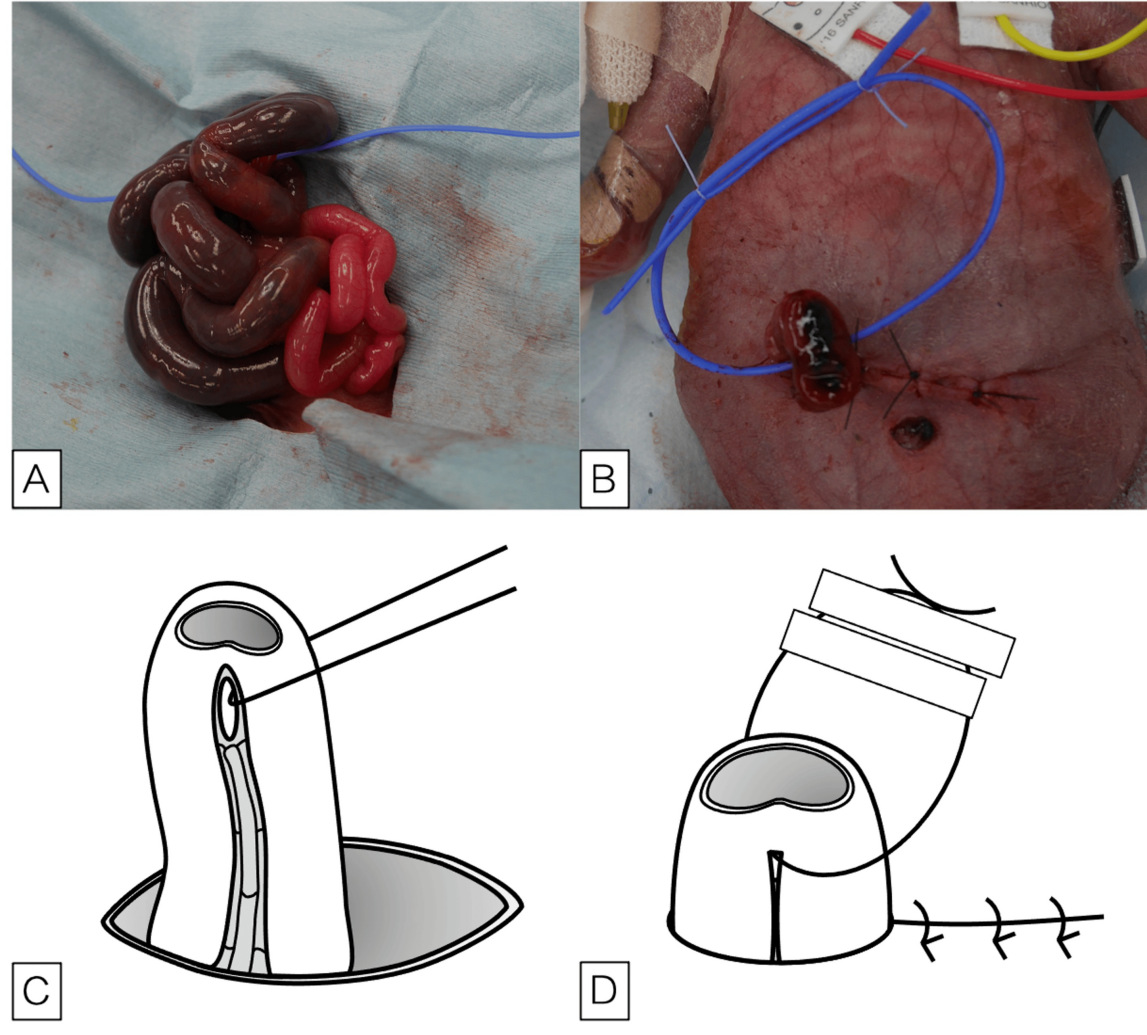
Intraoperative photographs and surgical schemas of sutureless loop enterostomy (A, C) After performing a detailed intraperitoneal examination, the vessel tape is passed through the mesentery just below the perforation of the small intestine. (B, D) The intestine is placed at the edge of the wound, and the peritoneum, muscular layer, and skin are closed. The vessel tape is attached to the skin with surgical tape.

**Figure 2:**
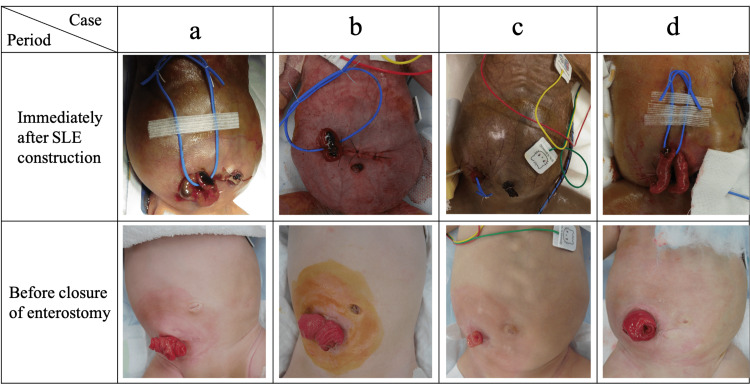
The appearance of four cases immediately after stoma construction and at the time of closure surgery in patients who underwent stoma construction with sutureless loop enterostomy (SLE). We present the pre- and post-operative conditions in four cases, case a to case d.

To perform SLE, the vessel tape is passed through the mesentery at the enterostomy site (Figure 1A, 1C), and the enterostomy stumps are placed at the edge of the laparotomy wound. In SLE, the length of bowel brought outside the abdominal cavity varies among cases. Ideally, we aim to bring out a length of bowel that is twice the diameter of the intestine to create the enterostomy. However, inflammation and adhesions resulting from bowel perforation often make it difficult to achieve sufficient bowel brought outside the abdominal cavity and ensure adequate exteriorized length. Therefore, in such cases, we create the enterostomy using the length of bowel that can be feasibly brought outside. The peritoneum and fascia are closed with 4-0 absorbable sutures, and the skin is closed with 4-0 or 5-0 nylon suture. The intestinal tract and skin are not fixed, but the vessel tape that was passed through the mesentery is fixed to the skin with surgical tape (Figure 1B, 1D). We present the appearance of cases immediately after stoma construction and at the time of closure surgery in patients who underwent stoma construction with SLE (Figure 2).

Immediately after the procedure, the stool is absorbed by the gauze placed around the enterostomy. If the stool is continuously discharged, the enterostomy may spontaneously invert and adhere to the skin. About one week after the operation, the vessel tape is removed because the enterostomy and the skin have adhered to each other. Furthermore, no specific measures are taken to prevent enterostomy desiccation. In most cases, additional measures are unnecessary because the incubator is humidified to protect the infant's skin. For infection control, stool is frequently removed, and the skin is cleansed with saline. A pouch is used as soon as bowel movements are observed to prevent desiccation and to ensure that stool does not come into direct contact with the wound site. Upon observing stool output from the enterostomy, enteral feeding with breast milk is initiated and gradually increased. If breast milk is not available or if there is a tendency for hard stools, elemental formulas may be used. If defecation from the enterostomy is inadequate, a Nelaton catheter may be inserted into the enterostomy to irrigate the intestine. If respiratory and circulatory dynamics are stable, a contrast enema can be performed antegrade via the mucous fistula about one week after the procedure. After confirming that there is no obstruction in the distal side of intestine, a feeding tube is placed into the distal side of the enterostomy, and the stool from the proximal side of the enterostomy is injected from there.

At our institute, we close the enterostomy when the patient weighs more than 2000 g. If malabsorption of nutrients is suspected or if the enterostomy prolapses, enterostomy closure is performed without waiting for weight gain. Before closure, an enema examination of the distal intestine is performed. For the closure procedure, a spindle-shaped incision is made in the skin around the enterostomy, and the enterostomy and skin are separated from the abdominal wall. An end-to-end anastomosis with single-layer, interrupted, 5-0 absorbable sutures is performed.

Surgical procedure of the conventional sutureless enterostomy (SE)

**Figure 3:**
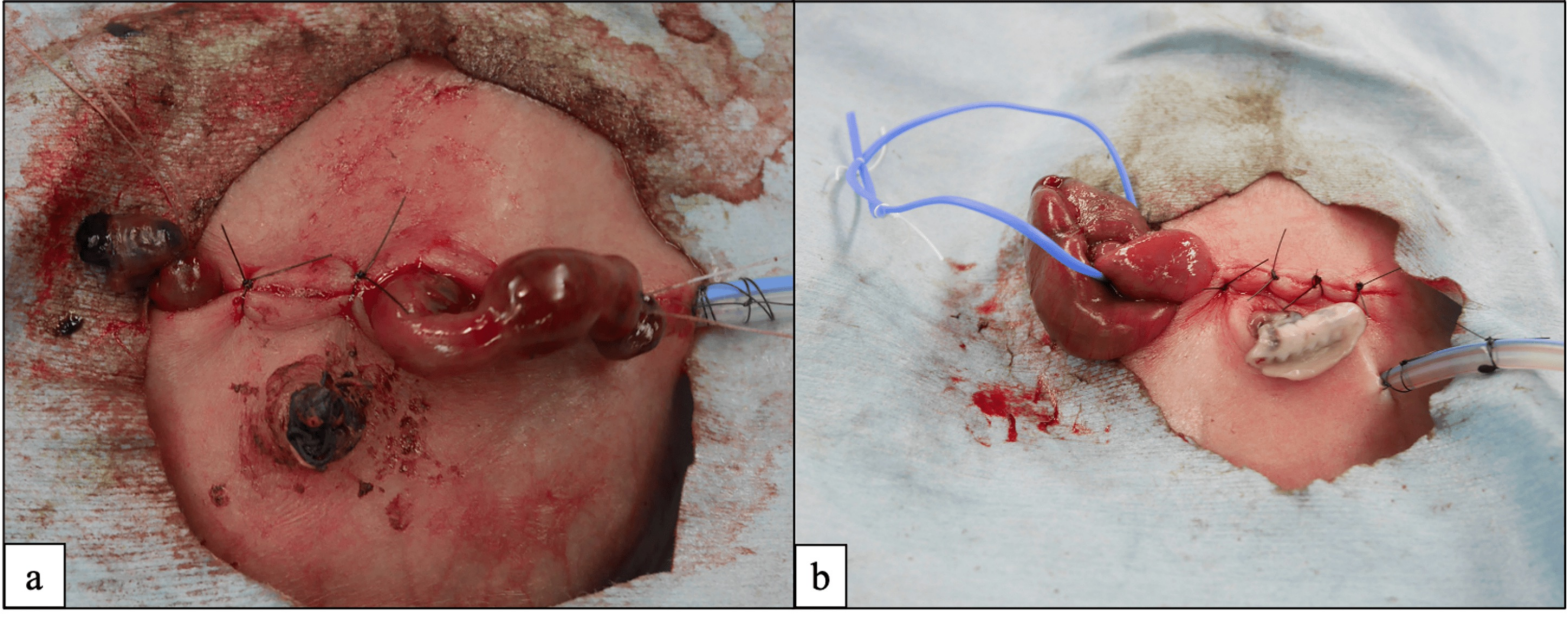
Appearance of enterostomy using the sutureless enterostomy (SE) procedure. One formed at both ends of the surgical site (a) and one formed using a double-barrel technique at one end of the surgical site (b).

At our institution, the SE procedure is performed similarly to the method reported by Ohashi et al. [6]. After laparotomy, the bowel is partially resected, including the perforated area, and the proximal and distal sides of the bowel are positioned at both ends of the abdominal incision. Additionally, we sometimes use a double-barrel technique to form an SE at one end of the wound. In both methods, vascular tapes are not used. Postoperative management is almost the same as for SLE. A photograph of the immediate postoperative state of SE is shown in Figure 3.

Statistical analysis

Statistical analysis was performed with the Chi-square test, the Mann-Whitney U test, and the Kruskal-Wallis test. All statistical analyses were conducted with commercial software (SAS OnDemand for Academics, SAS Institute, Inc., Cary, NC, USA). All P values were derived from two-tailed analyses, with significance accepted at a P value of less than 0.05. Because the comparison of the three groups was a multiple comparison, Bonferroni correction was performed and significance was accepted at a P value of less than 0.0167.

## Results

Demographics

We analyzed data of 32 very low and extremely low birth weight infants who underwent enterostomy. The mean ± standard deviation gestational age was 26.6 ± 4.09 weeks (range: 22-36 weeks), and birth body weight was 810.9 ± 314.58 g (range: 339-1456 g). The number of patients in each group was as follows: SLE, 12; SE, 6; and C, 14. Table 1 shows the underlying diseases.

**Table 1:** Underlying diseases in low birth weight or very low birth weight infants that underwent enterostomy FIP: focal intestinal perforation; NEC: neonatal necrotizing enterocolitis; MRI: meconium-related ileus

	Sutureless loop enterostomy group (n = 12)	Sutureless enterostomy group (n = 6)	Conventional enterostomy group (n = 14)
NEC	1 (8.3%)	4 (66.7%)	3 (21.4%)
FIP	6 (50.0%)	1 (16.7%)	2 (14.3%)
MRI	5 (41.7%)	1 (16.7%)	3 (21.4%)
other	0 (0%)	0 (0%)	6 (42.9%)

Comparison between the three groups

**Figure 4:**
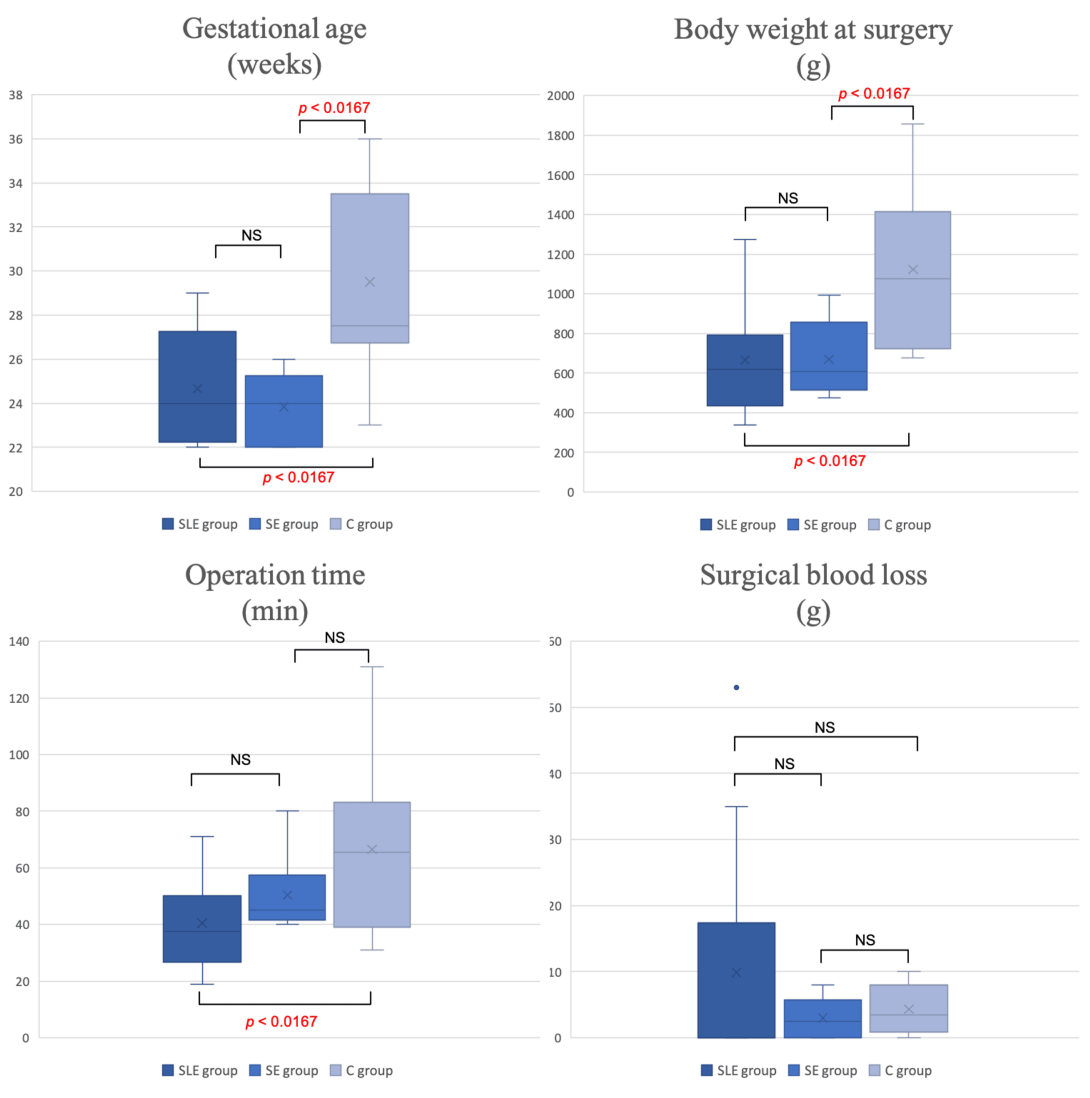
Box plot showing the patient characteristics and surgery-related items in the sutureless loop enterostomy, sutureless enterostomy, and conventional procedure groups. The presence or absence of a significant difference between each pair of groups was examined. Bonferroni correction was used to adjust the P values for multiple comparisons between the three groups. P values were calculated with the Mann-Whitney U test. C: conventional enterostomy; SE: sutureless enterostomy; SLE: sutureless loop enterostomy.

The comparison of the patient characteristics and surgery-related items in the three groups is shown in Table 2. Additionally, Figure 4 shows a box plot of each of the items from Table 2; pairwise comparisons were performed of items that showed a significant difference between the three groups. Sex and age at surgery and surgical blood loss were not different among the three groups, but gestational age (SLE: 24.7 ± 2.36 weeks; SE: 23.8 ± 1.57 weeks; and C: 29.5 ± 4.19 weeks) and body weight at surgery (SLE: 665.3 ± 285.25 g; SE: 669.8 ± 179.54 g; and C: 1123.2 ± 370.80 g) were significantly lower in the SLE and SE groups than in the C group. The operation time was not significantly different between the SLE and SE groups but was significantly shorter in the SLE group than in the C group (SLE: 40.4 ± 16.20 min; SE: 50.3 ± 13.67 min; and C: 66.5 ± 26.74 g).

**Table 2:** Patient characteristics and surgery-related items in three groups of low birth weight or very low birth weight infants that underwent enterostomy Data are shown as mean ± SD unless otherwise indicated. NS: not significant P-values were calculated using the Kruskal-Wallis test and the Chi-square test

	Sutureless loop enterostomy group (n = 12)	Sutureless enterostomy group (n = 6)	Conventional enterostomy group (n = 14)	P-value
Gender(female)	5 (41.7%)	2 (33.3%)	4 (28.6%)	NS
Birth body weight	681.4 ± 332.83	669.8 ± 179.54	982.4 ± 255.21	<0.05
Gestational weeks	24.7 ± 2.36	23.8 ± 1.57	29.5 ± 4.19	<0.05
Age at surgery (days)	12.8 ± 6.14	32.0 ± 45.81	19.9 ± 25.57	NS
Body weight at surgery (g)	665.3 ± 285.25	669.8 ± 179.54	1123.2 ± 370.80	<0.05
Operative time (min)	40.4 ± 16.20	50.3 ± 13.67	66.5 ± 26.74	<0.05
Bleeding (g)	9.9 ± 16.75	3.0 ± 3.16	4.3 ± 3.71	NS
Age at closure of enterostomy (days)	154.8 ± 32.83	154.2 ± 38.07	111.6 ± 42.50	<0.05
Body weight at closure of enterostomy (g)	2300.7 ± 397.87	2274.0 ± 365.60	2282.6 ± 684.74	NS

Table 3 shows the enterostomy-related complications in the three groups. Early complications occurred in one case each in the SLE and SE groups and in three cases in the C group. All cases with early complications required emergency enterostomy reconstructive surgery. Late complications occurred in eight cases in the SLE group, three in the SE group, and five in the C group. Emergency surgery was required in two cases in each of the three groups. In the remaining 10 cases with late complications, the enterostomy was closed by an elective operation. There was no significant difference in the number of early and late enterostomy-related complications between the three groups.

**Table 3:** Enterostomy-related complications in three groups of low birth weight or very low birth weight infants that underwent enterostomy All early complications required emergency surgery. NS: not significant P values were calculated using the Chi-square test for comparison between the three groups. ^a^Early complication: complications occurred less than 30 days after surgery ^b^Late complication: complications occurred 30 days after surgery

			Sutureless loop enterostomy group (n = 12)	Sutureless enterostomy group (n = 6)	Conventional enterostomy group (n = 14)	P value
Early complications^a^	Total		1 (8.33%)	1 (16.67%)	3 (21.43%)	NS
		Prolapse	1 (8.33%)	0 (0%)	2 (14.29%)	
		Necrosis	0 (0%)	1 (16.67%)	0 (0%)	
		Gastric perforation (due to postoperative intestinal obstruction)	0 (0%)	0 (0%)	1 (7.14%)	
Late complications^b^	Total		8 (66.67%)	3 (50.00%)	4 (28.57%)	NS
		Prolapse	5 (41.67%)	1 (16.67%)	3 (21.43%)	
		Enterostomy-site hernia	2 (16.67%)	0 (0%)	0 (0%)	
		Stenosis	1 (8.33%)	1 (16.67%)	1 (7.14%)	
		Enterostomy retraction	1 (8.33%)	0 (0%)	0 (0%)	
	Requiring emergency surgery		2 (16.67%)	2 (33.33%)	2 (14.29%)	NS

## Discussion

In the current study, we showed that SLE significantly shortened the operation time compared with the conventional approach. Moreover, we found no significant difference in the number of postoperative complications among the three different types of enterostomy. On the other hand, gestational age, birth body weight, and body weight at surgery were significantly lower in the SLE and SE groups than in the C group; this difference is thought to be due to the bias in selecting the surgical procedure, as SLE and SE were considered safer in infants with higher tissue vulnerability.

Neonatal surgeons have difficulty constructing enterostomies in very low and extremely low birth weight infants, therefore various surgical techniques, such as sutureless enterostomy, sutureless techniques using cyanoacrylate adhesives, T-tube enterostomies, and the fascial bridge technique between the stomas have been used [6, 8-10]. In these infants, sutureless enterostomy can prevent mechanical destruction of intestinal tissue and intestinal necrosis due to impaired blood flow because it does not require suturing of the thin and fragile intestinal tissue [7]. Similar sutureless procedures have been reported in adults [11].

SLE is a very simple procedure in which the intestine is pulled out of the peritoneal cavity. Therefore, the operation time is shorter than with conventional procedures, as shown in our study and in a previous study on sutureless enterostomy [4]. Furthermore, SLE is less invasive and shortens the duration of both the surgical and anesthesia procedures. Both SLE and SE are simple procedures that, in emergency situations such as intestinal perforation or obstruction, can be performed even by surgeons who are not proficient in operating on premature infants [4]. Due to their relative simplicity and applicability, these procedures have the potential to reduce delays in treatment and improve outcomes, even in settings where specialized neonatal surgical expertise may not be readily available.

Conventional enterostomy has a high incidence of enterostomy-related complications in low birth weight infants [1, 5, 9, 10]. These complications are related to the risks associated with suturing the intestinal wall, which is only 70 to 100 μm thick in extremely low birth weight infants and thus equivalent to the thickness of 5-0 and 6-0 sutures [6]. As the intestine is not fixed at all in SLE, we hypothesized that the rate of complications such as prolapse would increase; however, in our study, no significant increase in postoperative complications, including prolapse, was observed with SLE compared with SE and the conventional procedure. Although prolapses did occur after SLE (see Table 3), many of them did not require emergency surgery.

Causes of enterostomy prolapse include increased abdominal pressure and the degree of postoperative intra-abdominal adhesions [6, 12]. In particular, some studies suggested that sutureless enterostomy may increase the risk that complications such as prolapse would occur as infants grow [1, 4-6]. One study did find that the rate of enterostomy prolapse tended to increase over time [12]; however, age and weight at enterostomy closure were considered to have a greater influence on the occurrence of prolapse than whether or not the enterostomy procedure was sutureless or not. Furthermore, within the context of SE, the mesentery is not fixed, therefore makes it susceptible to inversion, thus the risk of prolapse is significantly increased [6]. In the present study, SLE did not show an increase in early enterostomy-related complications, including prolapse. Although the difference was not statistically significant, a relatively high frequency of prolapse of enterostomy was observed as a late complication. In extremely low birth weight infants, the incidence of enterostomy prolapse is high regardless of the surgical procedure [12]. SLE for premature infants born weighing less than 1000g or 1500g offers significant advantages by reducing surgical time and ensuring a safer early postoperative period.

It is considered difficult to completely prevent surgical site infection due to factors such as the general condition of the infant and potential intraoperative contamination [6]. However, surgical site infections in cases of neonatal bowel perforation are not very common [1]. Additionally, previous reports on sutureless enterostomy, similar to our approach, have established enterostomy at the laparotomy wound site and have managed to avoid surgical site infections through intraoperative decompression and wound protection [6]. In our cases as well, we have not encountered surgical site infections, suggesting that constructing an enterostomy at the wound site is viable and safe.

Some authors suggested that closure of an enterostomy in low birth weight infants should be performed 10 weeks after the first laparotomy [13] or after infants weigh 2.5 kg or more [12], and others found that closure within six weeks after the first laparotomy is not associated with additional complications [14]. Thus, no clear consensus has been reached yet on the optimal timing of closure. Although further studies are needed, studies to date indicate that it may be necessary to close an enterostomy, including an SLE, as early as possible in low birth weight infants to prevent late complications, including prolapse. Furthermore, there is a risk of late complications such as adhesions following enterostomy closure. It is necessary to conduct long-term follow-up and evaluation after enterostomy closure.

In cases requiring enterostomy, we routinely perform contrast studies on the distal side of the enterostomy in all patients to confirm the absence of any obstruction. Additionally, we insert a feeding catheter into the distal side of the enterostomy and inject stool that is expelled from the proximal side of enterostomy. This procedure is also referred to as “distal stoma refeeding”, and we refer to it as “recycling small intestinal contents” [15, 16]. Previous reports indicate that recycling small intestinal contents does not show a statistically significant contribution to weight gain or improved nutritional status [16]. This procedure is simple to perform and allows for verification that the distal segment of the intestine is patent. Additionally, stimulating the distal bowel may help prevent mucosal atrophy of the distal bowel and reduce the disparity in diameter between the proximal and distal bowel segments during stoma closure. In this study, we were unable to conduct a thorough examination because the underlying diseases requiring enterostomy and the locations of the stoma varied among the cases, making direct comparisons difficult. Further research is needed to evaluate the effectiveness of recycling small intestinal contents.

Several reports from Japan have described the sutureless enterostomy procedure [4, 6, 8], but it is not fully recognized internationally. Consequently, further research is required to determine whether sutureless enterostomy can become a standard procedure not only in Japan but also elsewhere.

Some limitations associated with this study warrant mention. First, this was a retrospective study with a small number of cases that was conducted at a single institution. The validity of the surgical procedure should ideally be evaluated by prospective multi-institutional joint research, although such research is difficult because each facility uses a different surgical procedure for SE. Secondly, this study excludes cases that resulted in death before the closure of the enterostomy. The causes of death in the excluded cases were often related to prematurity, such as intracranial hemorrhage, circulatory failure, or chromosomal abnormalities. The exclusion of these cases might have impacted the results of this study. Since various factors affect the prognosis of very low and extremely low birth weight infants, it is difficult to evaluate the impact of the two surgical procedures on mortality outcomes. In this study, such an evaluation was not possible. As more cases are accumulated in the future, it will be necessary to examine the impact of different surgical procedures of enterostomy for very low and extremely low birth weight infants on survival outcomes. Thirdly, SLE is a loop enterostomy, and its indication is limited to cases that do not require extensive intestinal resection. We do not currently consider SLE as an option for NEC cases requiring extensive intestinal resection. However, it may be applicable in cases with limited bowel damage (e.g., ≤5 cm), as creating a loop-shaped stoma at the perforation site could preserve intestinal length and reduce the risk of short bowel syndrome. Further studies are needed to explore its potential use in such cases. Last, the proportion of infants with NEC was lower in the SLE group than in other two groups, so the indications for SLE may have affected the results.

## Conclusions

In conclusion, the operation time was significantly shorter with SLE than with the conventional enterostomy procedure. SLE did not increase enterostomy-related complications compared with the conventional procedure and SE. Thus, in very low and extremely low birth weight infants whose general condition is poor and the surgical time needs to be shortened, SLE can be an option for enterostomy that does not require bowel resection.
